# Role of miR-9 in Modulating NF-κB Signaling and Cytokine Expression in COVID-19 Patients

**DOI:** 10.3390/ijms25168930

**Published:** 2024-08-16

**Authors:** Carla Prezioso, Dolores Limongi, Paola Checconi, Marco Ciotti, Jacopo M. Legramante, Carlo M. Petrangeli, Francesca Leonardis, Alfredo Giovannelli, Alessandro Terrinoni, Sergio Bernardini, Marilena Minieri, Cartesio D’Agostini

**Affiliations:** 1 Department for the Promotion of Human Sciences and Quality of Life, San Raffaele University, Via di Val Cannuta 247, 00166 Rome, Italy; dolores.limongi@uniroma5.it (D.L.); paola.checconi@uniroma5.it (P.C.); 2 Laboratory of Microbiology, IRCCS San Raffaele Roma, Via di Val Cannuta 247, 00166 Rome, Italy; 3 Unit of Virology, Tor Vergata University Hospital, 00133 Rome, Italy; marco.ciotti@ptvonline.it; 4 Department of Systems Medicine, University of Rome Tor Vergata, 00133 Rome, Italy; legraman@uniroma2.it; 5 Emergency Department, Tor Vergata University Hospital, 00133 Rome, Italy; carlo.petrangeli@ptvonline.it (C.M.P.); francesca.leonardis@uniroma2.it (F.L.); 6 Department of Surgical Sciences, University of Rome Tor Vergata, 00133 Rome, Italy; 7 Unit of Laboratory Medicine, Tor Vergata University Hospital, 00133 Rome, Italy; alfredo.giovannelli@gmail.com (A.G.); alessandro.terrinoni@uniroma2.it (A.T.); bernards@uniroma2.it (S.B.); 8 Department of Experimental Medicine, University of Rome Tor Vergata, 00133 Rome, Italy; d.agostini@med.uniroma2.it; 9 Laboratory of Microbiology, Tor Vergata University Hospital, 00133 Rome, Italy

**Keywords:** COVID-19, miRNA, miR-9, NF-κB, IκBα, IL-6, IL-1β, TNF-α

## Abstract

Coronavirus disease 2019 (COVID-19), caused by the SARS-CoV-2 virus, has had a significant impact on global health, with severe cases often characterized by a worsening cytokine storm. Since it has been described that the NF-κB signaling pathway, regulated by microRNAs, could play a pivotal role in the inflammatory response, in this study, the role of miR-9 in modulating NF-κB signaling and inflammatory cytokine expression in COVID-19 patients was investigated. This observational retrospective single-center study included 41 COVID-19 patients and 20 healthy controls. Serum samples were analyzed for miR-9, NF-κB, and IκBα expression levels using RT-PCR. The expression levels and production of pro-inflammatory cytokines IL-6, IL-1β, and TNF-α were measured using RT-PCR and ELISA. Statistical analyses, including correlation and regression, were conducted to explore relationships between these variables. COVID-19 patients, particularly non-survivors, exhibited significantly higher miR-9 and NF-κB levels compared to controls. A strong positive correlation was found between miR-9 and NF-κB expression (r = 0.813, *p* < 0.001). NF-κB levels were significantly correlated with IL-6 (r = 0.971, *p* < 0.001), IL-1β (r = 0.968, *p* < 0.001), and TNF-α (r = 0.968, *p* < 0.001). Our findings indicate that miR-9 regulates NF-κB signaling and inflammation in COVID-19. Elevated miR-9 levels in non-survivors suggest its potential as a severity biomarker. While COVID-19 cases have decreased, targeting miR-9 and NF-κB could improve outcomes for other inflammatory conditions, including autoimmune diseases, highlighting the need for continued research in this area.

## 1. Introduction

SARS-CoV-2, the causative agent of coronavirus disease 2019 (COVID-19) [1], has been the central focus of recent research due to its significant global health impact [2]. The molecular mechanisms underlying its pathogenesis involve intricate interactions between viral components and host cellular pathways, with the nuclear factor kappa B (NF-κB) signaling pathway playing a pivotal role [3].

NF-κB represents a family of transcription factors crucial in regulating a wide variety of cellular processes, including immune responses, inflammation, cell proliferation, differentiation, and survival [4]. Structurally, NF-κB exists as a dimer, primarily composed of proteins from the Rel family, including RelA (p65), RelB, c-Rel, p50/p105 (NF-κB1), and p52/p100 (NF-κB2) [5]. In inactive states, NF-κB dimers are sequestered in the cytoplasm through interactions with inhibitors known as IκB proteins. Upon activation by various stimuli, such as cytokines, free radicals, ultraviolet irradiation, and bacterial or viral antigens, IκB kinases (IKKs) phosphorylate IκB, leading to its ubiquitination and subsequent proteasomal degradation. This process liberates NF-κB dimers, allowing their translocation to the nucleus, where they bind to specific DNA sequences to regulate the transcription of target genes involved in inflammation, immune response, and cell survival [6].

Dysregulation of NF-κB has been implicated in various pathological conditions, including acute radiation syndrome [7], inflammatory and autoimmune diseases, cancer, metabolic disorders [8], and viral infections such as COVID-19. In the context of a SARS-CoV-2 infection, it is activated upon viral recognition, leading to the transcription of genes that promote an inflammatory response, which are crucial for combating the virus. However, this activation can also result in a cytokine storm, a severe response that has been implicated in many of the critical manifestations of COVID-19 [9]. It remains unclear why some individuals develop this hyper-inflammatory response while others do not. Among the possible mechanisms, it is hypothesized that genetic factors, variations in innate and adaptive immune responses, and pre-existing conditions may influence susceptibility to the cytokine storm. For instance, efficient regulation of immune signaling pathways such as NF-κB can prevent the inflammatory cascade in some individuals [10].

MicroRNAs (miRNAs) are small non-coding RNA molecules that play crucial roles in regulating gene expression by targeting mRNA and inducing translational repression or degradation [11]. Among these, miR-9 emerges as a significant regulator of the NF-κB signaling pathway. The intricate role of miR-9 in modulating NF-κB during viral infections underscores its dual functionality in both enhancing and suppressing immune pathways [12].

In the context of COVID-19, NF-κB plays a vital role in the cytokine storm, which is responsible for many of the severe manifestations of the disease. It has been suggested that miR-9 can positively regulate NF-κB activity by inhibiting the expression of SIRT1, a known negative repressor of NF-κB [13]. This suppression facilitates the phosphorylation and subsequent degradation of IκB. The decrease in IκB levels allows NF-κB to translocate to the nucleus and initiate the transcription of genes that promote inflammation, including the transcription of pro-inflammatory cytokines essential for the initial immune response against viral infections [14].

Conversely, miR-9 has also been found to negatively regulate NF-κB [15]. This occurs through its interaction with components upstream of the NF-κB pathway, where miR-9 targets and modulates proteins involved in NF-κB activation, leading to a suppression of NF-κB activity and thus attenuating the inflammatory response. Such regulation is essential in preventing the overactivation of immune responses, which can lead to tissue damage and exacerbate disease severity, as noted in studies examining miRNA profiles in COVID-19 patients [16].

The dualistic role of miR-9 in regulating NF-κB highlights the complex regulatory networks at play during SARS-CoV-2 infection and reflects the broader role of miRNAs in fine-tuning the immune response. In fact, by modulating NF-κB signaling, miR-9 not only contributes to the host’s ability to manage the inflammatory response effectively but also impacts the clinical outcomes and pathogenesis of COVID-19 patients.

Given the complexities involved in the regulation of NF-κB by miR-9, especially in the context of COVID-19, in this study, the mechanisms by which miR-9 could influence immune response modulation were investigated. Specifically, the expression levels of miR-9, NF-κB, and IκBα were measured. Moreover, the correlation and impact of miR-9 on the expression of pro-inflammatory cytokines IL-6, IL-1β, and TNFα, which are critical mediators in the inflammatory response associated with COVID-19 severity [17], were also assessed.

The evaluation of the potential role of miR-9 as a biomarker or therapeutic target to modulate the inflammatory response during SARS-CoV-2 infection could improve knowledge on COVID-19 clinical outcomes.

## 2. Results

### 2.1. Analysis of miR-9 Expression: Comparisons between COVID-19 Patients and Healthy Controls

MiR-9 levels were quantified in the serum of COVID-19 patients (*n* = 41), stratified into non-survivors (*n* = 23) and survivors (*n* = 18), and in the serum of subjects from the healthy control group (*n* = 20) (Table 1), using RT-PCR to ensure high specificity and sensitivity in detecting even minimal changes in miR-9 expression.

**Figure 1:**
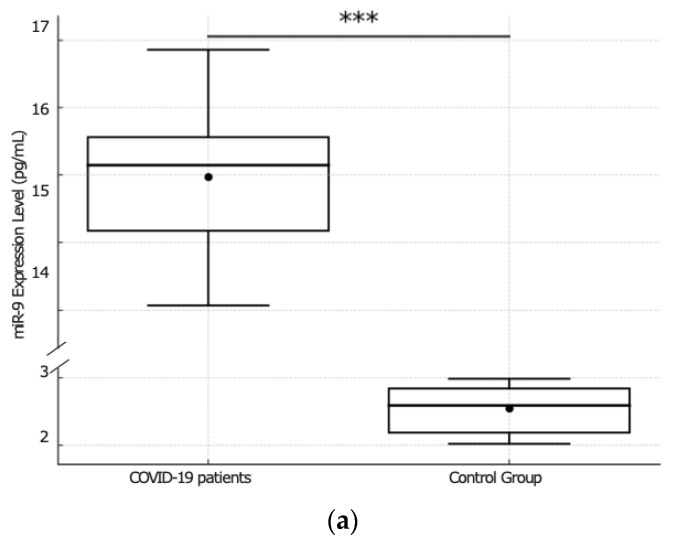

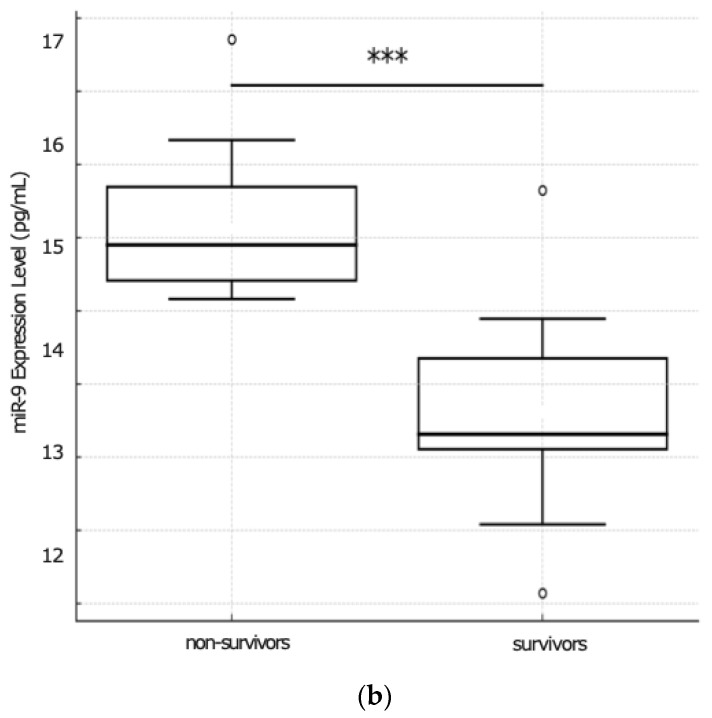
(**a**) Comparative analysis of miR-9 expression between COVID-19 patients and the control group. The figure shows a boxplot comparing the miR-9 expression levels (pg/mL) between COVID-19 patients (*n* = 41) and the control group (*n* = 20). COVID-19 patients exhibit significantly higher miR-9 levels compared to the control group (indicated by *** *p* < 0.001). (**b**) Comparative analysis of miR-9 expression between non-survivors and survivors COVID-19 patients. The boxplot compares the miR-9 expression levels (pg/mL) between two groups of COVID-19 patients: non-survivors (*n* = 23) and survivors (*n* = 18). Non-survivors exhibit significantly higher miR-9 levels compared to survivors (indicated by *** *p* < 0.001).

As shown in Figure 1, COVID-19 patients exhibited markedly higher median expression levels of miR-9 [15 pg/mL; interquartile range (IQR) 14.00–16.00 pg/mL] in contrast to the control group, in which the median expression level of miR-9 was 2.5 pg/mL with an IQR of 2.00–3.00 pg/mL (Figure 1a).

A comparative analysis between COVID-19 patients and control group revealed a difference in the expression levels of miR-9 (*p* < 0.001) (Figure 1a).

The next step was to investigate whether miR-9 expression levels differed among non-survivors and survivors. As shown in Figure 1b, non-survivors exhibited significantly higher median miR-9 expression levels [15 pg/mL; IQR 14.50–16.00] compared to survivors, who had a median expression level of 13 pg/mL with an IQR of 12.00–14.50. This difference in expression levels among non-survivors and survivors was statistically significant, as indicated by a *p* value < 0.001, underscoring a strong and non-random association.

To further understand the dynamics of miR-9 expression, age, and sex-specific analyses were conducted. These analyses revealed that the upregulation of miR-9 was more pronounced in older patients (>65 years) across both COVID-19 cohorts, with a notable gender disparity indicating higher levels in males.

### 2.2. NF-κB and IκBα Expression in the Serum of COVID-19 Patients and Healthy Controls

**Figure 2:**
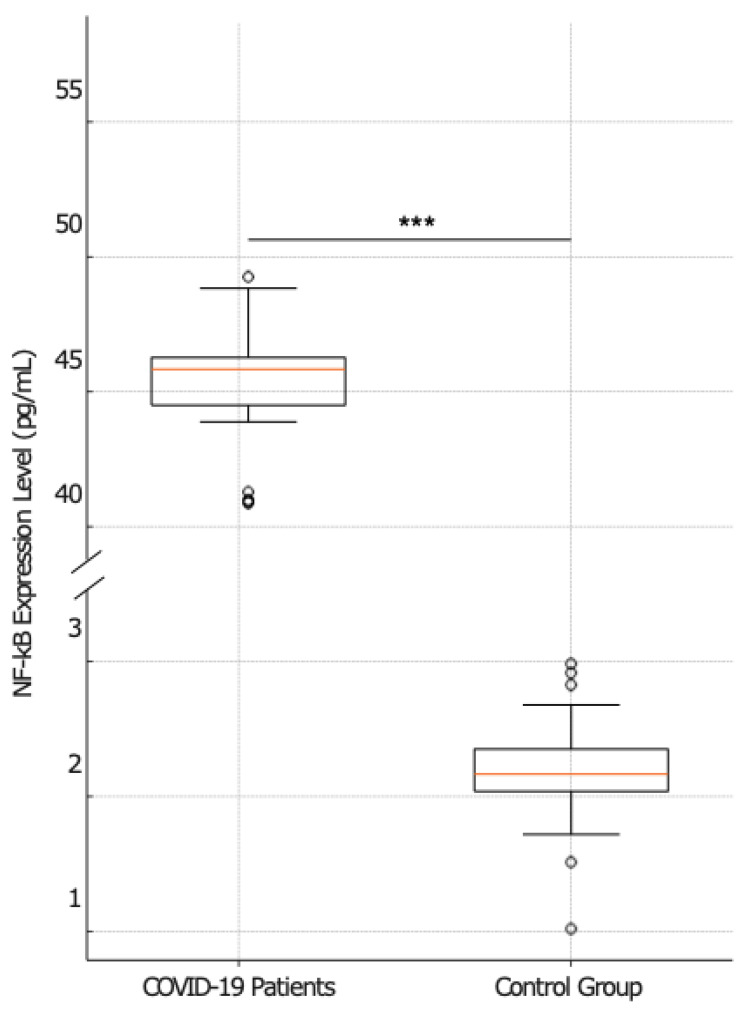
Comparative analysis of NF-κB expression between COVID-19-patients and the control group. The box plots illustrate the relative expression levels of NF-κB in the two groups. The left plot displays the distribution of NF-κB expression levels in patients with COVID-19 (*n* = 41), whereas the right plot shows the NF-κB expression levels in the control group (*n* = 20). The statistical analysis reveals a significant difference in NF-κB expression between the two groups (*p* < 0.001), marked by a line and three asterisks (***).

The serum level of NF-κB in COVID-19 patients (*n* = 41), stratified into non-survivors (*n* = 23),survivors (*n* = 18), and subjects that belonged to the control group (*n* = 20), was measured by RT-PCR. Specifically, the analysis of the NF-κB expression level in COVID-19 patients revealed a significant disparity when compared to a matched control group. The box plot (Figure 2) shows that COVID-19 patients exhibited a higher median expression level of NF-κB [15 pg/mL; IQR 44.50–49.50] in contrast to the control group, in which the median expression level of NF-κB was 13 pg/mL with an IQR of 1.50–2.50. The statistical significance of this discrepancy was confirmed by a *p* value < 0.001, indicating a robust and non-random association.

The correlation between patients’ age and the mean of NF-κB expression was assessed using Pearson’s correlation coefficient. The analysis revealed a correlation coefficient of 0.066, suggesting a very weak positive relationship. However, the *p* value associated with this correlation was 0.682, indicating that the correlation between age and NF-κB expression levels is not statistically significant. Differences in NF-κB expression between male and female patients were examined using an independent sample *t*-test. The test yielded a t-statistic of 1.30. With a *p* value of 0.201, the results do not support a statistically significant difference in the mean NF-κB expression levels between the two sexes.

Moreover, the level of IκBα, an inhibitor of NF-κB, in the serum of COVID-19 patients was also measured. Intriguingly, IκBα was undetectable in the COVID-19 cohort, whereas its presence was observable in the control group.

### 2.3. Detection of Proinflammatory Cytokines

**Figure 3:**
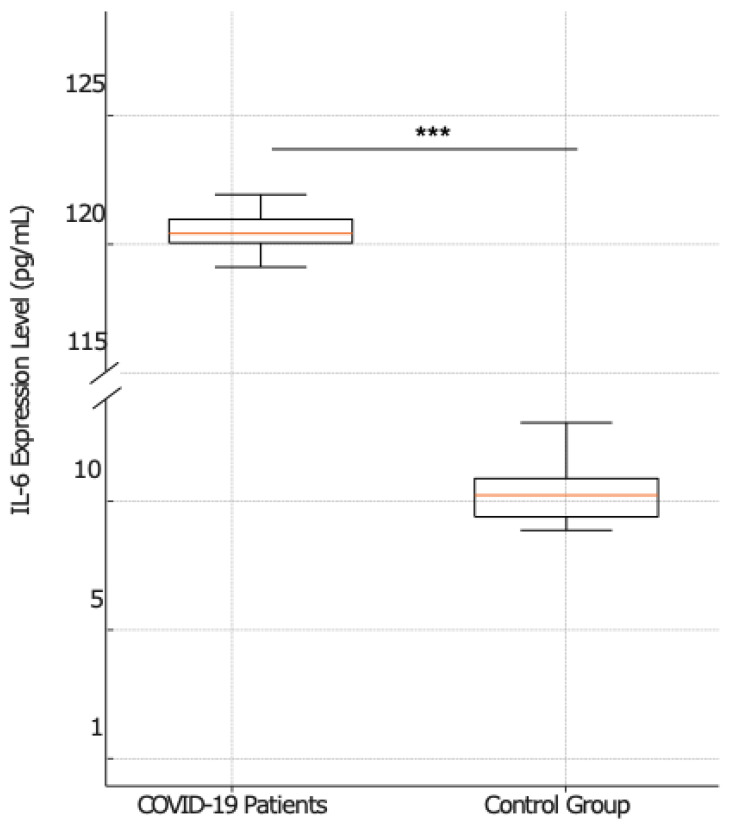
Comparative analysis of IL-6 expression between COVID-19-patients and the control group. The box plots display the mean of IL-6 expression levels among COVID-19 patients (left plot) (*n* = 41) and control group (right plot) (*n* = 20). The statistical analysis reveals a significant difference in IL-6 expression between the two analyzed groups (*p* < 0.001), marked by a line and three asterisks (***).

**Figure 4:**
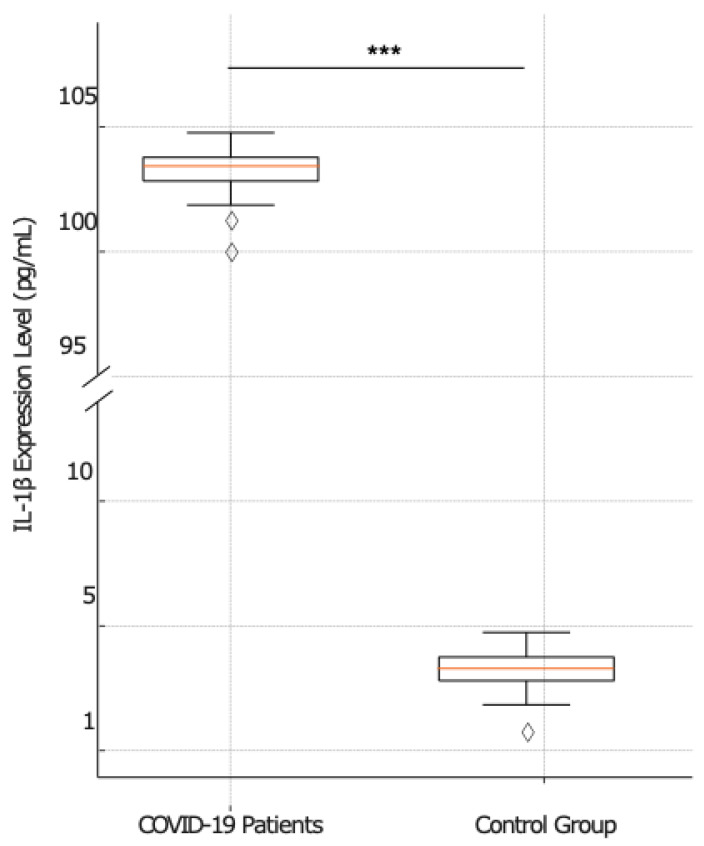
Comparative analysis of IL-1β expression between COVID-19-patients and the control group. The box plots show the distribution of mean IL-1β expression levels for COVID-19 patients (left) (*n* = 41) and the control group (right) (*n* = 20). The plots show a higher median and broader variance in IL-1β expression among COVID-19 patients compared to more consistent levels in the control group. The statistical analysis reveals a significant difference in IL-1β expression between the two analyzed groups (*p* < 0.001), marked by a line and three asterisks (***).

**Figure 5:**
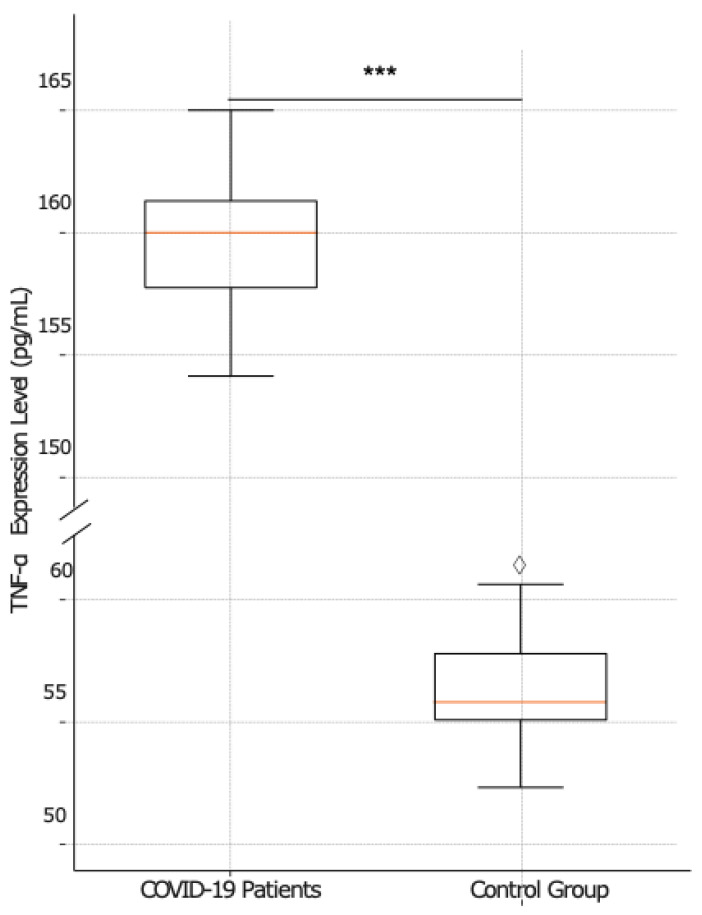
Comparative analysis of TNF-α expression between COVID-19-patients and the control group. The box plots show a higher median of TNF-α expression levels for COVID-19 patients (left) (*n* = 41) compared to control group (right) (*n* = 20). The statistical analysis revealed a significant difference in TNF-α expression between the groups (*p* < 0.0001), marked by a line and three asterisks (***) and indicating higher levels in the COVID-19 patients.

Since one of the functions of NF-κB is the regulation of some proinflammatory cytokine expression, NF-κB-mediated proinflammatory cytokine gene expression was also performed. The serum IL-6, IL-1β, and TNF-α expression levels were measured by RT-PCR in COVID-19 patients (*n* = 41), stratified into non-survivors (*n* = 23) and survivors (*n* = 18), and in the serum of subjects in the control group (*n* = 20). When the proinflammatory cytokine expressions were examined, a significant upregulation was observed in COVID-19 patients compared to the control group. (Figure 3, Figure 4 and Figure 5).

Specifically, as illustrated in Figure 3, COVID-19 patients demonstrated significantly higher median IL-6 expression levels [120 pg/mL; IQR 119.00–121.00] compared to the control group, which showed a much lower median IL-6 expression of 10 pg/mL, with the IQR (excluding outliers) ranging from 9.00 to 12.00. This difference between COVID-19 patients and the control group was statistically significant, with a *p* value < 0.001.

Further investigation into the potential correlation between IL-6 expression and patient age and sex was conducted. The relationship between age and IL-6 expression levels was analyzed using Pearson’s correlation coefficient, yielding a correlation coefficient of −0.111. This result indicates a weak inverse relationship between the two variables, but the associated *p* value of 0.488 suggests that this correlation does not reach statistical significance. This implies that age does not significantly influence IL-6 expression in COVID-19 patients. Additionally, differences in IL-6 expression levels between male and female patients were examined using an independent sample *t*-test, resulting in a t-statistic of 1.31 and a *p* value of 0.199. These findings indicate no statistically significant difference in IL-6 expression levels between the sexes, suggesting that sex, like age, does not significantly impact IL-6 expression within the cohort of COVID-19 patients analyzed.

The box plot in Figure 4 compares the IL-1β expression levels among COVID-19 patients and the control group. In COVID-19 patients, the median IL-1β expression is 103 pg/mL, with an IQR, excluding outliers, spanning from 101.00 to 105.00. In contrast, the control group shows a significantly lower median IL-1β expression of 3 pg/mL, with the IQR ranging from approximately 2.00 to 5.00. The difference in IL-1β expression levels between the two groups is highly significant, with a *p* value < 0.001.

To determine whether IL-1β expression in COVID-19 patients is significantly influenced by age or sex, two distinct statistical analyses were performed. Pearson’s correlation coefficient was used to assess the relationship between patient age and mean IL-1β expression, revealing a weak negative correlation (r = −0.259). Although this relationship was not statistically significant (*p* = 0.107), it suggests that age does not significantly affect IL-1β expression levels in this patient cohort. Furthermore, an independent *t*-test comparing IL-1β expression levels by sex resulted in a t-statistic of 0.061 and a *p* value of 0.952, indicating no significant differences in IL-1β expression between the sexes.

Figure 5 illustrates a box plot comparing TNF-α expression levels between COVID-19 patients and the control group. Among COVID-19 patients, the median TNF-α level is 160 pg/mL, with an IQR, excluding outliers, spanning from about 154.00 to 165.00. Conversely, the control group exhibits a considerably lower median TNF-α expression level of 56 pg/mL, with an IQR extending from around 52.00 to 60.00. The difference in TNF-α levels between the two groups is statistically significant, with a *p* value < 0.001.

The relationship between age and TNF-α levels was assessed using Pearson’s correlation coefficient, revealing a weak positive correlation (r = 0.18) that was not statistically significant (*p* = 0.259). This suggests that age does not significantly influence TNF-α expression levels in this cohort. Subsequently, a *t*-test comparing TNF-α expression levels between male and female patients yielded no statistically significant difference (*p* = 0.417), indicating that gender does not play a critical role in modulating TNF-α expression among COVID-19 patients.

### 2.4. Measurement of Cytokine Production in COVID-19 Patients vs. Control Group

**Figure 6:**
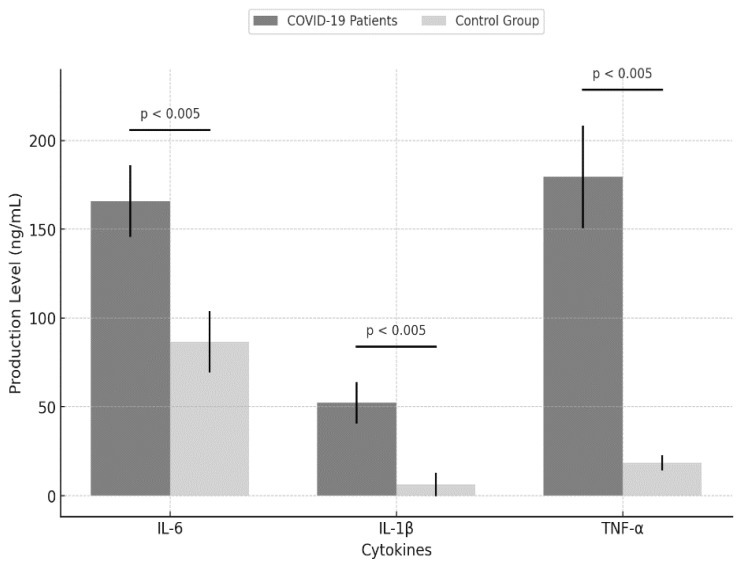
IL-6, IL-1β, and TNF-α production levels in COVID-19-patients vs. the control group. The bar graph compares the production of three cytokines (IL-6, IL-1β, and TNF-α) between COVID-19 patients (*n* = 41) and the control group (*n* = 20). The black lines above each pair of bars indicate that the differences are statistically significant, with *p* values < 0.005.

In order to confirm cytokine production, the Luminex Assay was performed to determine the levels of IL-6, IL-1β,and TNF-α. The results showed that COVID-19 patients had significantly higher levels of all three cytokines compared to the control group: IL-6 at 165.85 ng/mL (±20.13) vs. 86.68 ng/mL (±17.30); IL-1β at 52.30 ng/mL (±11.71) vs. 6.30 ng/mL (±6.61); and TNF-α at 179.54 ng/mL (±28.92) vs. 18.50 ng/mL (±4.25) (Figure 6).

### 2.5. Correlation Analysis between Serum miR-9, NF-κB and Inflammatory Factors in COVID-19 Patients

The relationship between the expression of miR-9, the transcription factor NF-κB, and the pro-inflammatory cytokines IL-6, IL-1β, and TNF-α in a cohort of COVID-19 patients was investigated.

**Figure 7:**
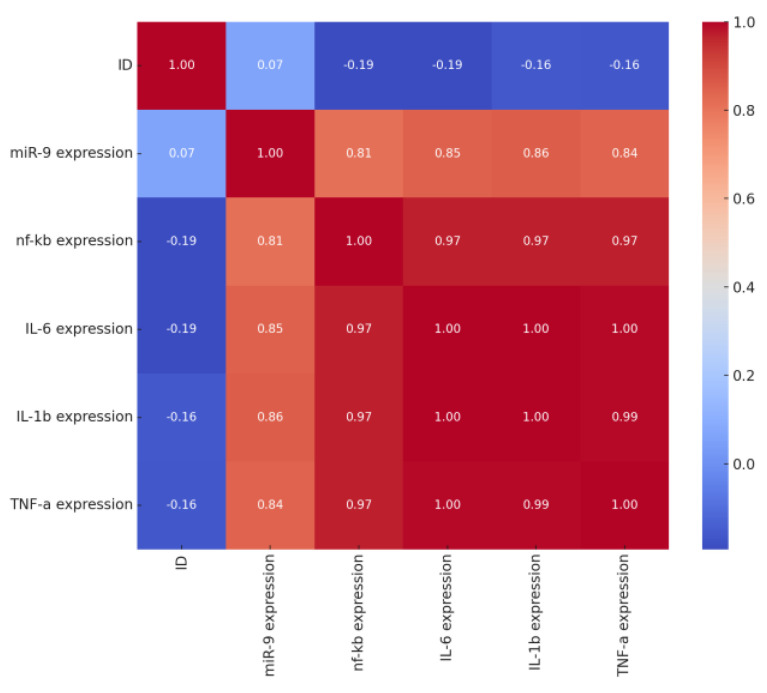
Correlation matrix of miR-9 expression, the NF-κB transcription factor, and the pro-inflammatory cytokines IL-6, IL-1β, and TNF-α in COVID-19 patients (*n* = 41). The correlation matrix shows the relationships between the expression of miR-9, NF-κB, and pro-inflammatory cytokines (IL-6, IL-1β, TNF-α). The correlation value is indicated inside each of the matrix cells, with a color scale ranging from blue (negative correlation) to red (positive correlation). There is a strong positive correlation between miR-9 and NF-κB expression (r = 0.813), as well as between NF-κB and each of the pro-inflammatory cytokines: IL-6 (r = 0.971), IL-1β (r = 0.968), and TNF-α (r = 0.968).

Specifically, the correlation matrix was employed to initially assess the strength and direction of the relationships in the expression levels of miR-9, NF-κB, and the pro-inflammatory cytokines IL-6, IL-1β, and TNF-α. This method provides a quantitative measure of how closely two variables are linearly related. The results revealed a strong positive correlation between miR-9 and NF-κB expression (r = 0.813, *p* < 0.001). Additionally, NF-κB showed very strong correlations with the expression levels of all analyzed pro-inflammatory cytokines: IL-6 (r = 0.971, *p* < 0.001), IL-1β (r = 0.968, *p* < 0.001), and TNF-α (r = 0.968, *p* < 0.001) (Figure 7).

### 2.6. Regression Analysis

**Figure 8:**
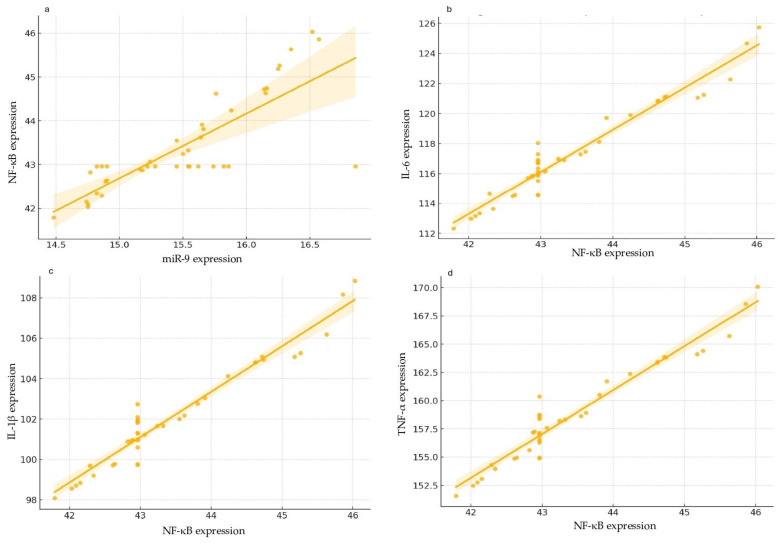
Linear regression analyses show the correlations among different gene expressions. In all the graphs, a clear positive linear correlation can be observed between the examined variables: miR-9 expression vs. NF-κB expression (**a**), NF-κB expression vs. IL-6 expression (**b**), NF-κB expression vs. IL-1β expression (**c**), and NF-κB expression vs. TNF-α expression (**d**). The distribution of data points around the regression line suggests a good fit of the linear regression model. The shaded areas represent the 95% confidence intervals for the regression lines. These intervals are relatively narrow, indicating that the estimates of the correlations are robust and precise despite the presence of some outliers.

The strong correlations obtained by the correlation matrix suggested potential regulatory relationships that merited further investigation through a regression analysis. The simple linear regression model was applied to determine whether variations in miR-9 expression could predict changes in NF-κB expression, establishing miR-9 as a potential upstream regulator. Results indicated that miR-9 expression is a significant predictor of NF-κB expression (R-squared = 0.661, *p* < 0.001). Specifically, each unit increase in miR-9 expression was associated with a 1.4774 unit increase in NF-κB expression (β = 1.4774, SE = 0.169, *p* < 0.001) (Figure 8a).

Moreover, a linear regression was also applied to determine whether NF-κB expression could predict the expression levels of pro-inflammatory cytokines IL-6, IL-1β, and TNF-α, thereby establishing its role as a mediator in the inflammatory response. The model showed that NF-κB is a strong predictor of IL-6 expression (R-squared = 0.943, *p* < 0.001). An increase of one unit in NF-κB expression corresponded to a 2.7765 unit increase in IL-6 expression (β = 2.7765, SE = 0.135, *p* < 0.001) (Figure 8b).

The regression analysis also indicated that NF-κB significantly predicts IL-1β expression (R-squared = 0.937, *p* < 0.001). An increase of one unit in NF-κB expression was associated with a 2.7201 unit increase in IL-1β expression (β = 2.7201, SE = 0.133, *p* < 0.001) (Figure 8c).

Finally, the linear regression analysis revealed that NF-κB is a significant predictor of TNF-α expression (R-squared = 0.937, *p* < 0.001). An increase of one unit in NF-κB expression corresponded to a 3.8854 unit increase in TNF-α expression (β = 3.8854, SE = 0.161, *p* < 0.001) (Figure 8d).

### 2.7. Multiple Linear Regression Analysis and Power Analysis

The multiple linear regression analysis and power analysis were applied to strengthen the results of previous analyses and to ensure the statistical validity of the conclusions. These techniques provide a better understanding of the combined effect of miR-9 and NF-κB on the expression of pro-inflammatory cytokines.

**Figure 9:**
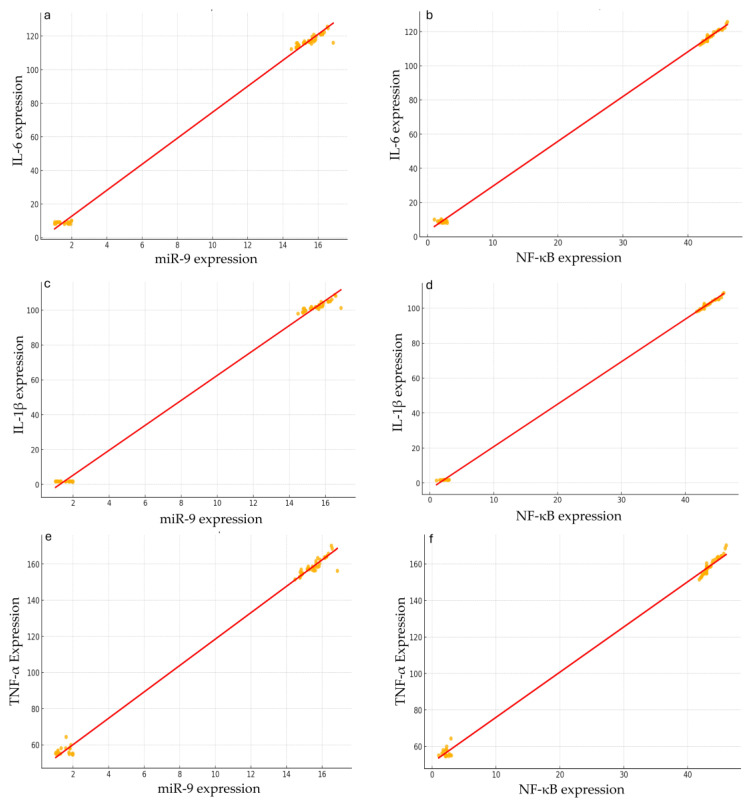
Expressions of IL-6, IL-1b, and TNF-α in relation to miR-9 and NF-κB. The graphs demonstrate the correlation between cytokine expression (IL-6, IL-1β, and TNF-α) and the expressions of miR-9 and NF-κB in COVID-19 patients (*n* = 41). (**a**,**b**) Show IL-6 expression plotted against miR-9 and NF-κB, respectively. The regression line highlights a strong positive correlation with an R^2^ value of 0.9996, indicating that almost all the variation in IL-6 expression is explained by miR-9 and NF-Κb. (**c**,**d**) Show IL-1β expression plotted against miR-9 and NF-κB. A positive correlation is observed, with an R^2^ value of 0.9997. (**e**,**f**) show TNF-α expression plotted against miR-9 and NF-κB. The regression indicates a positive correlation, with an R^2^ value of 0.9984. Points are only shown at the beginning and end of the regression lines to simplify the visualization of the combined effect of the two factors, emphasizing the slope and intercept of the model.

The results obtained from the multiple linear regression analysis confirm that both miR-9 and NF-κB have a significant positive effect on the expression of pro-inflammatory cytokines IL-6, IL-1β, and TNF-α. The coefficients for miR-9 and NF-κB were highly significant (*p* < 0.001 for most correlations), indicating that miR-9 and NF-κB substantially contribute to the increased expression of these cytokines. Specifically, for IL-6 expression, the coefficient for miR-9 was 1.302 (*p* < 0.001), indicating a strong positive relationship (Figure 9a). Similarly, the coefficient for NF-κB was 2.185 (*p* < 0.001), further highlighting its substantial impact on IL-6 expression (Figure 9b).

Regarding IL-1β expression, miR-9 showed a coefficient of 0.780 (*p* < 0.007), demonstrating a significant but slightly weaker positive effect compared to IL-6 (Figure 9c). On the other hand, NF-κB had a coefficient of 2.167 (*p* < 0.001), indicating a very strong and significant influence on IL-1β expression (Figure 9d).

For TNF-α expression, miR-9 exhibited a coefficient of 1.987 (*p* < 0.005), underscoring a notable positive effect (Figure 9e). NF-κB, with a coefficient of 1.804 (*p* < 0.001), also showed a significant positive relationship with TNF-α expression (Figure 9f).

The power analysis indicated that the sample size used in this study is sufficient to detect significant effects, with an estimated sample size of three participants for the analyses of IL-6 and IL-1β and four participants for the analysis of TNF-α.

## 3. Discussion

SARS-CoV-2, responsible for COVID-19 [1], has garnered extensive research attention due to its profound impact on global health [2]. The molecular pathogenesis of this virus involves complex interactions between viral elements and host cellular mechanisms, with the NF-κB signaling pathway being critically involved.

To assess the role of MiR-9 in regulating NF-κB during a SARS-CoV-2 infection, this study examined its impact on IκBα activity. Moreover, the correlation and impact of miR-9 on the expression of pro-inflammatory cytokines IL-6, IL-1β, and TNFα, which are critical mediators in the inflammatory response associated with COVID-19 severity [16], were also assessed.

The results suggest that miR-9 plays a significant role in regulating the inflammatory response following SARS-CoV-2 infection. The evidence showing increased miR-9 expression in non-surviving COVID-19 patients compared to survivors and healthy controls indicates that miR-9 could be used as a biomarker for disease severity. This increase is particularly relevant in older male patients, suggesting that demographic factors influence miR-9 expression.

This demographic-specific expression could explain the observed variations in COVID-19 severity. Houshmandfar et al. (2021) also noted that miR-223, another miRNA involved in regulating inflammation, exhibits varied expression levels based on demographic factors, underscoring the complexity of miRNA regulation in inflammatory diseases [18].

The significantly greater activation of NF-κB in COVID-19 patients compared to the control group, especially in non-survivors, implies that NF-κB plays a crucial role in disease progression. The undetectable levels of IκBα in COVID-19 patients suggest that SARS-CoV-2 infection may cause degradation or suppression of IκBα, leading to NF-κB activation. This finding highlights an altered regulatory mechanism in the NF-κB signaling pathway, which is critical for the innate immune response [19]. Also, in a study by Amini-Farsani et al. (2021), the role of miR-9 in regulating NF-κB and other inflammatory pathways in COVID-19 was highlighted, suggesting that targeting miRNAs could offer therapeutic benefits [20].

The increased expression of pro-inflammatory cytokines IL-6, IL-1β, and TNF-α in COVID-19 patients compared to the control group confirms the sequence of events following NF-κB activation. These cytokines are known for their role in promoting intense inflammatory responses, which can lead to tissue damage and disease exacerbation [7]. The high production of these cytokines in severe COVID-19 patients suggests that NF-κB activation is a key factor in inducing an intense inflammatory response during SARS-CoV-2 infection [17].

The results of this study indicate that miR-9 could be a useful biomarker for assessing COVID-19 disease severity and monitoring disease progression. Additionally, regulating miR-9 and targeting the NF-κB signaling pathway could represent promising therapeutic strategies to modulate the inflammatory response in COVID-19 patients. The potential degradation or suppression of IκBα and the unchecked activation of NF-κB observed in patients suggest that restoring IκBα levels or directly inhibiting NF-κB could attenuate inflammation and improve clinical outcomes.

The correlation matrix and linear regression were used to validate the potential role of miR-9 in regulating the expression of NF-κB and pro-inflammatory cytokines. These results provide a robust foundation for understanding the regulatory dynamics between miR-9, NF-κB, and pro-inflammatory cytokines, offering insights into the molecular mechanisms driving inflammation in the COVID-19 cohort. Moreover, the multiple linear regression analysis and power analysis were applied to strengthen the results of the previous analyses and ensure the statistical validity of the conclusions. These techniques allow for a better understanding of the combined effect of miR-9 and NF-κB on the expression of pro-inflammatory cytokines.

Our results demonstrate that miR-9 significantly regulates the expression of NF-κB, which in turn significantly influences the expression of the pro-inflammatory cytokines IL-6, IL-1β, and TNF-α.

This upregulation of NF-κB by miR-9 is crucial in driving the inflammatory processes that are characteristic of severe COVID-19 infections. A study on pediatric pneumonia showed that miR-20a, similar to miR-9, promotes inflammation by activating the NF-κB signaling pathway. Overexpression of miR-20a in lung cells increased the levels of pro-inflammatory cytokines IL-6 and TNF-α, along with the activation of NF-κB [21].

The strong correlation between miR-9, NF-κB, and cytokine expression observed through multiple linear regression analysis and power analysis provides a clear and detailed view of the combined effect of miR-9 and NF-κB on the expression of pro-inflammatory cytokines. These results confirm that miR-9 and NF-κB are critical regulators of inflammation in COVID-19 patients. The statistical robustness of the results ensures that the conclusions drawn are reliable and valid.

Understanding the regulatory mechanisms involving miR-9 and NF-κB opens up new patterns for therapeutic interventions. Targeting miR-9 to modulate NF-κB activation and the subsequent production of pro-inflammatory cytokines could provide significant benefits in treating severe COVID-19 cases. This approach is supported by findings that show how modulation of miRNA levels can affect inflammatory responses. For instance, studies on miR-146a mimics, which inhibit NF-κB-driven inflammation, suggest potential therapeutic strategies that could be applied to miR-9 [22].

This study’s findings support a regulatory model where miR-9 enhances the inflammatory response by activating NF-κB and subsequently increasing the expression of pro-inflammatory cytokines. This insight into the molecular mechanisms underlying inflammation in COVID-19 not only enriches our understanding but also highlights potential therapeutic targets. miR-9 could serve as a valuable biomarker and therapeutic target. Modulating miR-9 levels could represent a promising strategy to control inflammation and improve clinical outcomes in COVID-19 patients.

## 4. Materials and Methods

### 4.1. Study Design

This study was an observational, retrospective, single-center analysis. Forty-one COVID-19 patients admitted to the Emergency Department and then transferred to the Intensive Care Unit of the University Hospital Tor Vergata (Rome, Italy) during the second pandemic wave in Italy (March–June 2021) were enrolled. The 41-patient cohort was composed of 24 males and 17 females, aged from 29 to 86 years, stratified into two groups: survivors and non-survivors. Twenty subjects (10 males and 10 females) without COVID-19 were enrolled as a control group. Hypertension, cardiovascular diseases, and diabetes represented the most frequent comorbidities in both populations (Table 1). A diagnosis of COVID-19 was made by a positive real-time reverse transcription polymerase chain reaction (RT-PCR) taken from nasopharyngeal swabs and through radiological imaging. The design of the study was evaluated and approved by the local Ethics Committee at Tor Vergata University Hospital (approval number 87/20; 26 May 2020) and carried out according to the Declaration of Helsinki. Written informed consent was waived because of the rapid spread of this infectious disease.

### 4.2. Blood Sample Collection

Specimens from 41 COVID-19 patients and from 20 subjects chosen as the control group were recovered from blood samples used for routine laboratory testing. Upon arrival at the laboratory, blood samples were centrifuged at 2000 × *g* for 10 min at 4 °C to obtain serum samples. After routine analyses, specimens were aliquoted and stored at −80 °C until further use.

### 4.3. RNA Isolation

Total RNA, including miRNA, was extracted from previously aliquoted serum samples using the miRNeasy Mini Kit (Qiagen, Hilden, Germany), according to the manufacturer’s instructions. Extracted RNA was stored at −80 °C, and quality and concentration were measured with a NanoDrop spectrophotometer (Thermo Fisher Scientific, Waltham, MA, USA).

### 4.4. Reverse Transcription-PCR (RT-PCR)

Reverse transcription-PCR (RT-PCR) was carried out using a SYBR green one-step RT-PCR kit according to the manufacturer’s instructions and an AMPLilab™ Real-Time PCR System (Adaltis, Rome, Italy). The thermal profile used to measure the expression of miRNA and mRNA was as follows: 10 min at 55 °C, followed by 1 min at 95 °C, and 40 cycles of 10 sec at 95 °C, 30 sec at 53 °C, and 60 sec at 60 °C. The experimental data was calculated using the 2^−ΔΔCt^ method and normalized using GAPDH. Table 2 details the primers used in this study.

### 4.5. Enzyme-Linked Immune Sorbent Assay (ELISA)

The levels of various serum cytokines, including IL-6, IL-1β, and TNFα, were measured by ELISA using a Luminex Assay (Bio-Rad, Hercules, CA, USA) and an ELISA Kit (Enzo Life Sciences, Farmingdale, NY, USA) according to the manufacturer’s instructions [23]. Colors intensity was measured at 450 nm wavelength. The sensitivity of each cytokine was 1.03 ng/mL, 10.07 pg/mL, and 1.52 ng/mL, respectively.

### 4.6. Statistical Analysis

Statistical analyses were performed using SPSS software (https://www.ibm.com/analytics/spss-statistics-software, accessed on 1 August 2024). Before conducting comparative analyses, the Shapiro–Wilk test was used to assess the normality of the distribution of data for miR-9, NF-κB, IκBα, the pro-inflammatory cytokines (IL-6, IL-1β, TNF-α), and age. The choice of statistical tests was based on the results of these normality tests. For data that followed a normal distribution (*p* > 0.05 from the Shapiro–Wilk test), parametric tests were employed. Specifically, an independent sample *t*-test was used to compare the means of two independent groups (e.g., male vs. female, age groups if age data were normally distributed), whereas Pearson’s correlation coefficient was used to assess the linear correlation between two normally distributed continuous variables.

For data that did not follow a normal distribution (*p* ≤ 0.05 from the Shapiro-Wilk test), non-parametric tests were applied as follows:

The Mann–Whitney U test was used to compare differences between two independent groups when the data do not meet the assumptions of normality (e.g., age groups if age data are not normally distributed); Spearman’s Rank Correlation Coefficient was used to evaluate the strength and direction of the monotonic relationship between two continuous or ordinal variables.

For regression analyses, a simple linear regression was used to determine whether variations in miR-9 expression could predict changes in NF-κB expression, assuming the normality of residuals, whereas a multiple linear regression was applied to evaluate the combined effects of miR-9 and NF-κB on cytokine expression, assuming the normality of residuals.

A power analysis was conducted to ensure that the sample size was adequate to detect significant effects, enhancing the robustness of the conclusions drawn from the statistical analyses. All tests were two-tailed, and a *p* value of <0.05 was considered statistically significant.

## 5. Conclusions

The COVID-19 pandemic has almost ended, circulation of the virus has significantly reduced, and there is a minimal incidence of patients with severe or life-threatening conditions. However, in the results of this study offer new therapeutic perspectives for COVID-19 patients and could potentially be applied to conditions characterized by intense inflammation, such as autoimmune diseases and other viral infections. Implementing therapeutic strategies based on the regulation of miR-9 and NF-κB signaling could, in fact, reduce inflammation and significantly improve the clinical management of a wide range of respiratory diseases, although further research is needed to validate these applications.

In conclusion, what we learned from the inflammatory response to COVID-19 opens new strategies for the treatment of various pathological conditions, underscoring the importance of continued research in this field to enhance global health.

## Figures and Tables

**Table 1:** Demographic, epidemiological, and clinical data of COVID-19 patients and control group.

	Overall COVID-19	Survivors	Non-Survivors	Control Group NO COVID-19
N	41	18	23	20
Age				
Years, mean (SD)	68.78 ± 12.34	64.89 ± 11.08	71.83 ± 12.86	63.2 ± 12.01
Sex				
Male, N; Female, N	M, 24; F, 17	M, 12; F, 6	M, 12; F, 11	M,10; F,10
Comorbidities				
Hypertension, N (%)	22 (53.66%)	6 (54.55%)	16 (53.33%)	8 (40%)
Cardiovascular diseases, N (%)	6 (14.63%)	1 (9.09%)	5 (16.67%)	5 (25%)
Diabetes, N (%)	4 (9.76%)	2 (18.18%)	2 (6.67%)	4 (20%)
Others, N (%)	9 (21.95%)	2 (18.18%)	7 (23.33%)	3 (15%)

**Table 2:** Primer sequences.

Gene	Forward Sequence	Reverse Sequence
miR-9	5′-TCTTTGGTTATCTAGCTGTATGA-3′	5′-GTGCAGGGTCCGAGGT-3′
IL-6	5′-CTCTGCAAGAGATCCATCCA-3′	5′-GACAGGTCTGTTGGGAGTGG-3′
IL-1β	5′-GCTGAAAGCTCTCCAC-CTCA-3′	5′-GTTGGGATCCACACTCTCC-3′
TNF-α	5′-GCCTCTTCTCATTCCTGCTTG-3′	5′-CTGATGAGGGAGGCCATT C-3′
NF-κB	5′-GAAATTCCCTGATCCAGACAAAAAC-3′	5′-ATCACTTCAATGGCCTCTGTGTAG-3′
IκBa	5′-CAT CTC CAC TCC GTC CTG-3′	5′-GCA CCC AAA GTC ACC AAG-3′
GAPDH	5′-AGGTCGGTGTGAACGGATTTG-3′	5′-TGTAGACCATGTAGTTGAGGTCA-3′
U6	5′-CTCGCTTCGGCAGCACA-3′	5′-AACGCTTCACGAATTTGCGT-3′

## Data Availability

Data is contained within the article.
